# Quantitative Proteomic Analysis of Alligator Weed Leaves Reveals That Cationic Peroxidase 1 Plays Vital Roles in the Potassium Deficiency Stress Response

**DOI:** 10.3390/ijms21072537

**Published:** 2020-04-06

**Authors:** Li-Qin Li, Cheng-Cheng Lyu, Jia-Hao Li, Chuan-Yin Wan, Lun Liu, Min-Qiu Xie, Rui-Jie Zuo, Su Ni, Fan Liu, Fu-Chun Zeng, Yi-Fei Lu, Li-Ping Yu, Xue-Li Huang, Xi-Yao Wang, Li-Ming Lu

**Affiliations:** College of Agronomy, Sichuan Agriculture University, Chengdu 611130, China; chengchengLyu@163.com (C.-C.L.); 18889589812@139.com (J.-H.L.); wchy512@163.com (C.-Y.W.); ll2950385@163.com (L.L.); 15178695573@163.com (M.-Q.X.); Z18629501176@163.com (R.-J.Z.); ns13@163.com (S.N.); Liufantl2006@163.com (F.L.); zengfuchun78@163.com (F.-C.Z.); sarklu@126.com (Y.-F.L.); ylplzh@163.com (L.-P.Y.); hxueli1983@163.com (X.-L.H.); wxyrtl@163.com (X.-Y.W.)

**Keywords:** alligator weed, proteomics, leaf, cationic peroxidase, potassium

## Abstract

Alligator weed is reported to have a strong ability to adapt to potassium deficiency (LK) stress. Leaves are the primary organs responsible for photosynthesis of plants. However, quantitative proteomic changes in alligator weed leaves in response to LK stress are largely unknown. In this study, we investigated the physiological and proteomic changes in leaves of alligator weed under LK stress. We found that chloroplast and mesophyll cell contents in palisade tissue increased, and that the total chlorophyll content, superoxide dismutase (SOD) activity and net photosynthetic rate (PN) increased after 15 day of LK treatment, but the soluble protein content decreased. Quantitative proteomic analysis suggested that a total of 119 proteins were differentially abundant proteins (DAPs). KEGG analysis suggested that most represented DAPs were associated with secondary metabolism, the stress response, photosynthesis, protein synthesis, and degradation pathway. The proteomic results were verified using parallel reaction monitoring mass spectrometry (PRM–MS) analysis and quantitative real-time PCR (qRT-PCR)assays. Additional research suggested that overexpression of cationic peroxidase 1 of alligator weed (ApCPX1) in tobacco increased LK tolerance. The seed germination rate, peroxidase (POD) activity, and K^+^ content increased, and the hydrogen peroxide (H_2_O_2_) content decreased in the three transgenic tobacco lines after LK stress. The number of root hairs of the transgenic line was significantly higher than that of WT, and net K efflux rates were severely decreased in the transgenic line under LK stress. These results confirmed that ApCPX1 played positive roles in low-K^+^ signal sensing. These results provide valuable information on the adaptive mechanisms in leaves of alligator weed under LK stress and will help identify vital functional genes to apply to the molecular breeding of LK-tolerant plants in the future.

## 1. Introduction

Alligator weed (*Alternanthera philoxeroides*) belongs to the family Amaranthaceae, order Caryophyllales, subclass Caryophyllidae. It is an amphibious and perennial herb weed of Amaranthaceae. It is native to South America and has spread to many countries and regions such as North America, Australia, Africa, and Asia [1]. Alligator weed was initially planted as terrestrial forage in China in the 1930s. This plant mainly adopts asexual propagation and can adapt to different growth environments. The rhizome survives easily and forms new plants in suitable environments. Stems are hollow and buoyant, and the floating mats can expand over surfaces of all types of waterways, which clogs drainage canals, reduces water flow, and affects farmland irrigation [2]. Furthermore, alligator weed often forms a single dominant community and endangers the diversity of local plants. Previous researchers showed genome-wide DNA methylation and epigenetic regulation contribute to its high adaptability to harsh environments [3]. Under global warming conditions, the harm to native species from alligator weeds will increase at higher latitudes because of high competitiveness [4], so it has become one of the world′s recognized malignant weeds [5].

Potassium (K) plays crucial roles in many physiological and biochemical processes in plants, such as photosynthesis, protein synthesis, enzyme activation, osmotic regulation, ion homeostasis, and stomatal movement [6]. Therefore, K starvation leads to plant growth arrest, nitrogen and sugar imbalance, and increased susceptibility to abiotic and biotic stress. Although K is the seventh most abundant element in the earth’s crust [7], soluble K (K^+^) levels are very low in agricultural soils. In natural environments, low-K conditions are often transient, and therefore, plants have developed mechanisms to adapt to short-term LK stress. One important aspect of plants is that they employ both high-affinity and low-affinity K^+^ transport systems to sense K^+^ concentrations in the soil, and the high-affinity system functions when K^+^ concentrations are below 0.2 mM. The low-affinity system operates at K^+^ concentrations above 0.5 mM; thus, these transporters transport K^+^ across various membranes into various tissues to help plants survive under LK stress [8,9]. Moreover, it is clear that CBL–CIPK23 complexes modulate AtAKT1 and OsAKT1 activities by phosphorylation to absorb more K^+^ under LK stress in *Arabidopsis* and rice [10,11]. Alligator weed is found to have a strong potassium accumulation ability [12], and alligator weed root transcriptome analysis results have suggested that the expression levels of many transcription factors, kinases, and transporters change to adapt to LK stress [13]. Because gene expression can be regulated at the transcriptional, posttranscriptional, translational, and posttranslational levels, plant responses to LK are well controlled by LK-responsive proteins; thus, the identification of many functional or regulatory proteins is a fundamental step toward understanding their molecular mechanisms in response to stress in plants.

In recent years, the use of proteomics has been a cutting-edge approach to investigate all the proteins whose expression patterns change and how plants respond to abiotic stresses at the proteomic level. Leaves are the main organs for photosynthesis; changes in protein abundance in leaf cells alter the regulation of photosynthesis, further inducing changes in energy metabolism and primary/secondary metabolism. In the leaves of ramie, 27 differentially expressed proteins (DEPs) were analyzed after six days of LK treatments using two-dimensional electrophoresis (2-De) proteome analysis. These DEPs are involved in photosynthesis, energy metabolism, primary metabolism, signal transduction, protein synthesis, etc. [14]. Using quantitative proteomic analysis, Zeng et al. reported a total of 288 DEPs between leaves of low-K^+^- and normal K^+^-treated barley plants, and further analysis suggested that, compared with sensitive barley, LK-tolerant barley is highly capable of altering ion homeostasis and deploying an antioxidant defense system under LK stress [15]. In recent research, 55 DEPs were found in alligator weed leaves after LK stress using 2-DE techniques [16]. Because many hydrophobic proteins, proteins with a very high or very low molecular weight, and highly acidic or highly basic proteins are often lost during 2D-gel separation, in this study, a quantitative proteomic analysis was conducted to determine protein abundance changes after LK stress, and ApCPX1 was found to play positive roles in LK signal sensing. The aim of this study was to discover the molecular basis of potassium tolerance of alligator weed leaves, which provides a valuable resource for future plant molecular breeding.

## 2. Results

### 2.1. Effect of LK Stress on the Physiology of Alligator Weed Leaves

**Figure 1:**
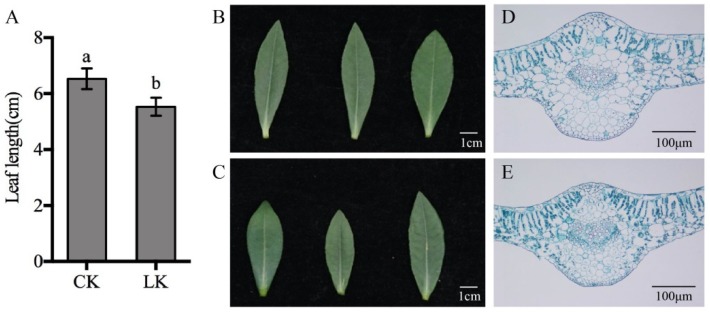
Morphological and microstructural characteristics of alligator weed leaves under different potassium conditions. Note (**A**) statistical results of leaf length; (**B**) leaves from CK; (**C**) leaves from LK; (**D**) leaf microstructure under CK conditions; (**E**) leaf microstructure under LK conditions. Values are the mean ± SD (*n* = 3), and different letters indicate significant differences (*p* < 0.05) between the treatments, CK indicates control, LK indicates potassium deficiency.

**Figure 2:**
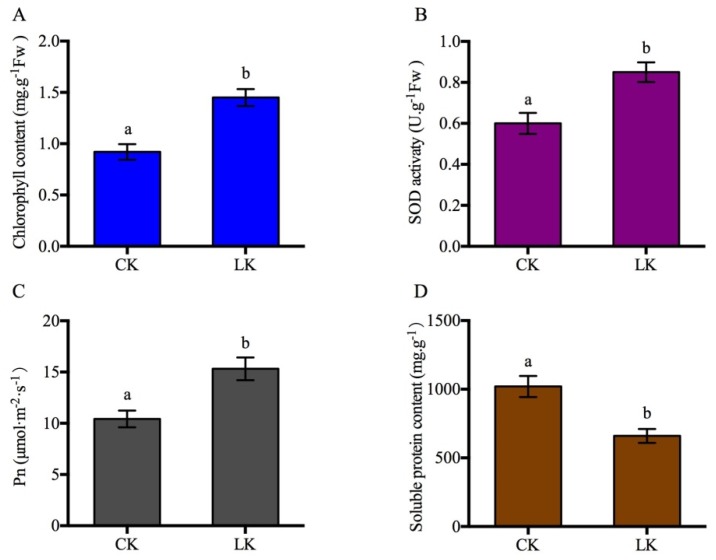
Leaf physiological parameters were analyzed. Note (**A**) total chlorophyll content; (**B**) SOD activity; (**C**) net photosynthetic rate; (**D**) soluble protein content. Values are the mean ± SD (*n* = 3), and different letters indicate significant differences (*p* < 0.05) between the treatments, CK indicates control, LK indicates potassium deficiency.

To determine the physiological responses of the leaves after LK treatment, leaves from the same position of the plants were collected. The results showed that the leaf length of the LK-treated plants was shorter than that of the control (CK) (Figure 1A–C), and the chloroplast and mesophyll cell contents in the palisade tissue increased according to paraffin section analysis (Figure 1D,E). The chlorophyll content, SOD activity, net photosynthetic rate (PN), and soluble protein content were then measured. The total chlorophyll content, SOD activity, and PN increased after treatment (Figure 2A–C), and the soluble protein content decreased (Figure 2D). This indicated that the size, internal structure, and physiological state of leaves changed after 15 days of treatment.

### 2.2. Subcellular Location and Protein Domain Analysis of DAPs

**Figure 3:**
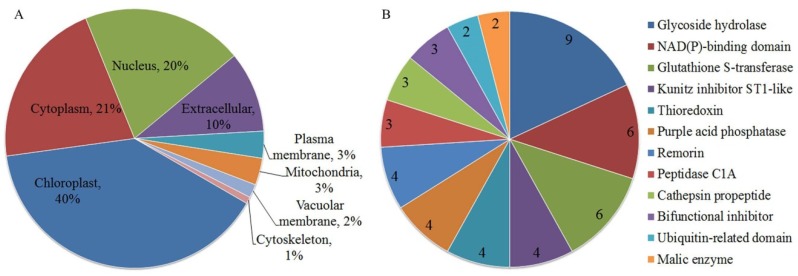
Analysis results of subcellular localization and protein domain. Note (**A**) subcellular localization analysis was derived from TargetP1.1 prediction; (**B**) protein domain analysis.

We employed tandem mass tags (TMT) and liquid chromatograph-mass spectromete(LC–MS/MS) to characterize the proteomic profiles of the leaves. The proteomic data showed that LK dramatically changed the protein abundance in the leaves. A change of more than 1.2-fold or a cut-off of less than 0.83-fold (*p* < 0.05) was considered statistically significant. Among quantifiable proteins, a total of 119 proteins were regarded as differentially abundant proteins (DAPs). Among these proteins, 63 were up-regulated, and 56 were down-regulated (Table S1). The subcellular locations of 119 DAPs were predicted by Target P1.1 software. The results suggested that 47 proteins showed chloroplast localization, of which 21 were increased; 25 proteins showed cytoplasm localization, of which 15 were increased; and 24 proteins showed nucleus localization, of which 14 were increased; these proteins constituted the top three groups. Localization in the plasma membrane, mitochondrion, vacuolar membrane, and cytoskeleton was the lowest, with protein numbers of 4, 4, 2, and 1, respectively (Figure 3A). According to the protein domain analysis, the top three protein groups were glycoside hydrolases, NAD(P)-binding domain-containing proteins, and glutathione S-transferases, of which 9, 6, and 6 were mapped, respectively, and the next 4 Kunitz inhibitor ST1-like proteins were all down-regulated (Figure 3B).

### 2.3. GO and KEGG Analyses of DAPs

**Figure 4:**
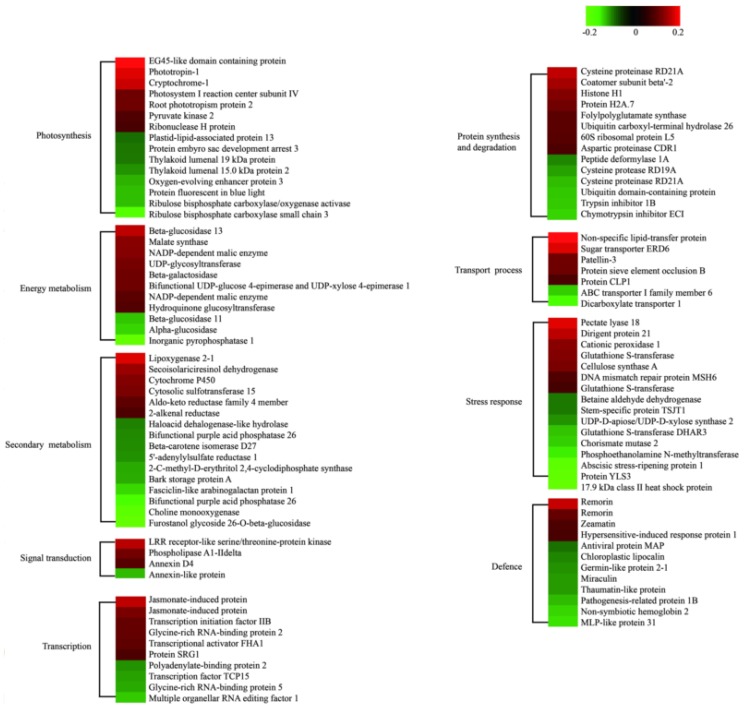
Enrichment results of DAPs by KEGG analysis. Note red indicates increased protein abundance; green indicates decreased protein abundance.

All DAPs were annotated and classified according to their biological process (BP), molecular function (MF), and cellular component (CC) according to the Gene Ontology (GO) database. The primary categories of BP were metabolic processes, single-organism processes, and cellular processes; the most abundant categories of CC were membrane, macromolecular complex, and cell, and the prominent MF categories were catalytic activity, binding, and molecular function regulator (Figure S1). The biological metabolic pathways related to the 119 DAPs were subsequently investigated using Kyoto Encyclopedia of Genes and Genomes (KEGG) analysis. The results suggested that most represented DAPs were associated with secondary metabolism and the stress response (both were associated with 16 DAPs), photosynthesis (15 DAPs), and protein synthesis and degradation (14 DAPs), while the fourth and fifth categories were associated with defense pathways and energy metabolism, respectively; each group included 12 to 11 DAPs (Figure 4).

### 2.4. Complementation of the Proteomic Results via PRM–MS and qRT-PCR

**Figure 5:**
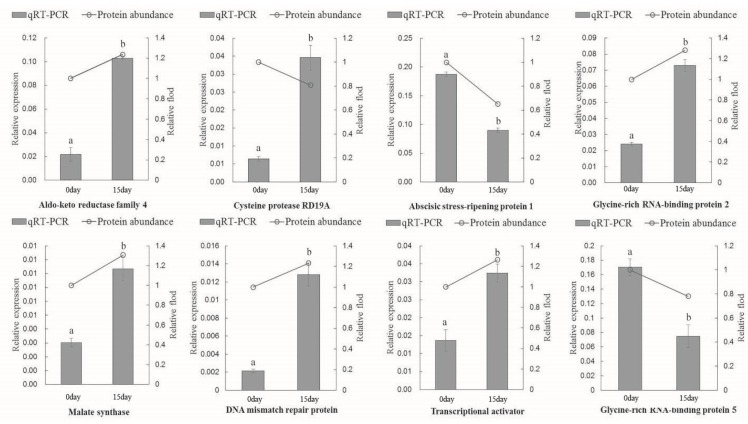
Complementation of the proteomic results by qRT-PCR. Note values are the mean ± SD (*n* = 3), and different letters indicate significant differences (*p* < 0.05) between the treatments, 0 day indicates control, 15 day indicates LK treatment for 15 day.

Six DAPs were chosen for quantification by PRM–MS analysis to verify the proteomic results (Table 1). As this assay required the signature peptide of the target protein to be unique, we selected only proteins with a unique signature peptide sequence for the PRM analysis. Six DAPs were identified in the leaves: malate synthase, cysteine proteinase RD19A, betaine aldehyde dehydrogenase, antiviral protein MAP, major latex protein 31, and choline monooxygenase. In general, the fold changes of these detected proteins were in agreement with the findings of the proteomic analysis. Our PRM analysis illustrated that the proteomic results were credible for subsequent analysis. A total of eight proteins were randomly selected to complement the accuracy of the proteomic data via qRT-PCR. There were seven gene expression patterns that showed the same tendencies as those of the expressed proteins: aldo-keto reductase family 4, abscisic stress-ripening protein 1, glycine-rich RNA-binding protein 2, malate synthase, DNA mismatch repair protein, transcriptional activator, and glycine-rich RNA-binding protein 5. Only one cysteine protease, RD19A, showed an opposite expression pattern (Figure 5).

### 2.5. Overexpression of Cationic Peroxidase 1 in Tobacco Increases LK Tolerance

**Figure 6:**
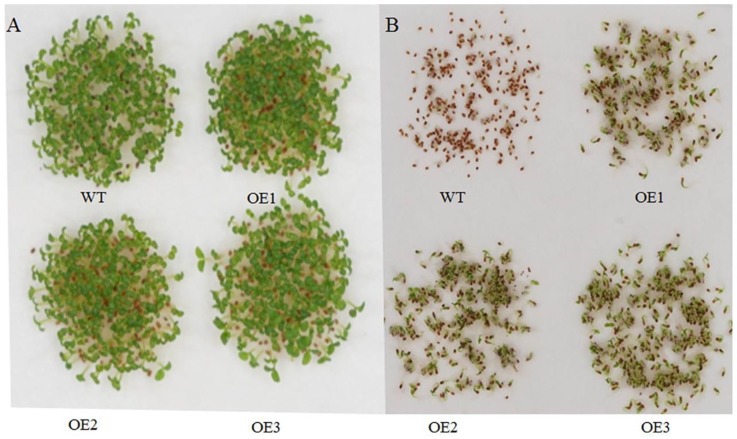
LK stress response of *ApCPX1* transgenic tobacco lines. Note (**A**) Morphological observation of WT and transgenic lines under CK conditions; (**B**) Morphological observation under LK conditions. WT indicates non-transgenic tobacco; OE1, OE2, and OE3 indicate transgenic tobacco lines.

**Figure 7:**
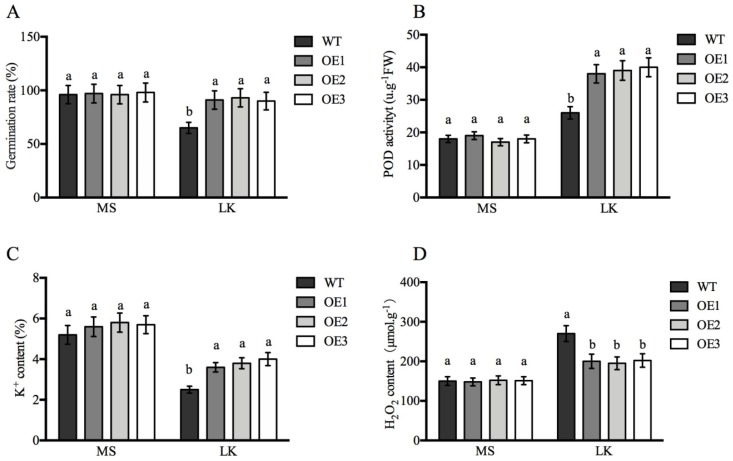
The physiological parameter analysis of wild-type and *ApCPX1* transgenic tobacco lines under MS and LK conditions. Note (**A**) germination rate; (**B**) POD activity; (**C**) K^+^ content; (**D**) H_2_O_2_ content. Values are the mean ± SD (*n* = 3), and different letters indicate significant differences (*p* < 0.05) between the treatments. MS indicates MS medium, LK indicates LK medium (containing 10 μm K^+^).

**Figure 8:**
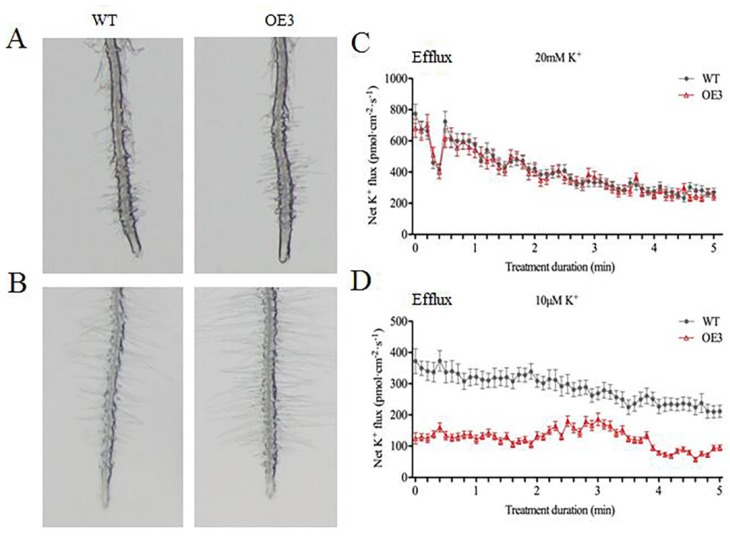
Root hair number and net K flux rates in roots of *ApCPX1* transgenic tobacco lines. Note (**A**) morphology of root hairs under CK conditions; (**B**) morphology of root hairs under LK conditions; (**C**) net K flux rate analysis under CK conditions; (**D**) net K flux rate analysis under LK conditions. Positive value indicates K efflux.

In *Arabidopsis*, overexpressing POD can improve *AtHAK5* expression in response to LK stress [17]. Proteomic analysis suggested only one cationic peroxidase 1 (ApCPX1) increased in abundance in the leaves after LK treatment in our study; we then cloned this gene and overexpressed it in tobacco to determine its function in LK stress. Additional research suggested the expression level of *ApCPX1* in three transgenic tobacco lines was higher than that of non-transgenic tobacco (WT) (Figure S2), and the germination rate of the transgenic tobacco seeds (OE1, OE2, OE3) was similar in the WT and the three transgenic lines under normal growth conditions (Figure 6A); however, the germination rate of the transgenic lines was obviously higher than that of the WT after 10 days of LK treatment (Figure 6B). Further results suggested that the seed germination rate, POD activity, and K^+^ content all increased (Figure 7A–C), while the H_2_O_2_ content decreased in the three transgenic tobacco lines compared with the WT under LK stress (Figure 7D). The observation experiment of root hair showed that there was no difference in the number of root hairs between the WT and transgenic tobacco OE3 line growing on MS medium for 10 d, but the root hairs of the OE3 line were significantly higher in number than WT after LK treatment (Figure 8A,B). Next, further research showed net K flux rates in roots between WT and the OE3 line were similar under 20 mM K external conditions, and net K efflux rates were severely decreased in the OE3 line compare to WT under LK stress (Figure 8C,D). These results confirmed that the overexpression of ApCPX1 increased the LK stress tolerance of transgenic tobacco.

## 3. Discussion

### 3.1. GO Term and KEGG Pathway Analyses of DAPs under LK Stress

GO enrichment analysis of DAPs identified was performed to detect physiological processes in response to LK stresses. The result showed that there were 185 DAPs significantly enriched in three main categories including cellular component, molecular function, and biological process (Table S2). The thylakoid lumenal 19 kDa protein, from membrane part (GO:0044425) in the cellular component, plays an essential role in plant photosynthesis. In *Arabidopsis*, loss of function of this protein causes the interruption of the photosystem II (PSII) repair process under changing light conditions, leading to decreased photosynthesis efficiency [18]. In our study, this protein was significantly down-regulated, indicating that decreased photosynthetic efficiency might be one of the responses of alligator weed in adaption to low potassium stress. Hydrolase activity (acting on glycosyl bonds) (GO:0016798) was enriched in molecular function. With respect to this term, there were two β-glucosidase isoforms significantly differentially expressed, β-glucosidase 11 was down-regulated while βeta-glucosidase 13 was up-regulated. The physiological function of β-glucosidase is mainly related to abiotic stress response, such as dehydration and salt. The transient over-expression of this gene resulted in the accumulation of antioxidant flavonols and improved tolerance to abiotic stresses in *Nicotiana benthamiana* [19]. In our study, the different expression pattern of these two isoforms suggested β-glucosidase maybe also involved in alligator weed low potassium stress response and the complexity of this response mechanism. The carbohydrate metabolic process (GO:0005975) was enriched in the biological process. In plants, carbohydrate metabolism provides energy and many essential cofactors and substrates for other metabolic processes. Pyruvate kinase (PK) is a key enzyme in carbon metabolism and the glycolytic pathway. Previous studies showed that down-regulated expression of PK altered the growth of transgenic tobacco plants [20]; rice OsPK2 is involved in endosperm starch synthesis, compound granule formation, and grain filling [21]. In our study, PK2 was significantly up-regulated, suggesting changes in the carbohydrate metabolic process could be an important mechanism to cope with LK stress in plants [22].

KEGG analysis showed that there were 9 metabolism pathways that were significantly enriched. Among them, secondary metabolism, photosynthesis, and the protein synthesis and degradation pathway were the top three, in which 16, 15, and 14 DAPs, respectively, were involved (Figure 4). A previous study reported that loss of function of folylpolyglutamate synthetase (FPGS) led to decreased lignin content and enhanced cell wall digestibility in *Arabidopsis* [23]. Thus, in our study, the high expression of FPGS in the alligator weed leaves could improve the lignin content in the cell wall in order to protect cells under LK stress. Corbin et al. reported that some direct proteins (DIRs) can be involved in secondary cell wall biosynthesis and plant defense in flax [24]. In our study, one DIR was up-regulated, underlying the universal roles DIRs play in plant abiotic stress response.

Phototropins (Phots), blue light (BL) receptors, are autophosphorylating serine/threonine protein kinases that play a critical role in the response to light quality, duration, and intensity, including mediating chloroplast relocation, stomatal opening, and leaf flattening and positioning [25]. In *Arabidopsis*, phototropin-1 (Phot1) triggers hypocotyl phototropism at both low and high blue light intensities. Previous studies suggest that Phot1 mediates first and second positive phototropism [26] and its activation in membrane microdomains is mediated by light induction [27]. Root phototropism 2 (RPT2) is a signal transducer associated with phototropin 1 and is involved in the phototropic response and stomatal opening in *Arabidopsis* [28]. In our study, in line with the up-regulation of Phot1 and RPT2 under LK stress, chloroplast number and PN were increased after treatment in alligator weeds leaves (Figure 1, Figure 2). As photosynthesis serves as the major energy source of plants and is directly affected by LK stress, we hypothesized that high expression of Phot1 and RPT2 could optimize photosynthesis in response to LK stress [29]. Ribosomal proteins (RPs) are well known for their universal roles in forming and stabilizing the ribosomal complex to mediate protein synthesis. The high expression of *RPL23A* resulted in enhanced drought and salt stress tolerance in rice seedlings by increasing the fresh weight, root length, and proline and chlorophyll contents [30]. One 60S ribosomal protein was up-regulated in our study, suggesting its roles in response to LK stress in alligator weed leaves. Ubiquitination is an important sort of posttranslational modification of proteins across all eukaryotes and is also involved in a wide range of abiotic stresses [31]. Ubiquitin carboxyl-terminal hydrolases (UCHs) play essential roles in recycling Ub and reversing the action of Ub conjugation. Previous studies have revealed that UCHs are crucial for the ubiquitination and degradation of the AUX/IAA family of repressors, which is the key step in auxin signaling [32]. In addition, a recent report suggested that UCH-L1 plays a key role in regulating reactive oxygen species ROS levels by deubiquitination in human umbilical vein endothelial cells [33]. Therefore, the up-regulated expression of UCHs in our study indicates their role in auxin and ROS signaling in alligator weed leaves under LK stress.

### 3.2. DAPs Related to Signal Transduction and Transcription

Serine threonine kinase receptors (RSTKs) interact with other proteins to modify a wide array of proteins by phosphorylation. Ouyang *et al.* reported increased SOD activity and a reduced H_2_O_2_ content in OsSIK1-overexpressing plants, and subsequent studies suggest that OsSIK1 plays important roles in salt and drought stress tolerance in rice through the activation of the antioxidative system [34]. In our study, an RSTK was up-regulated in the leaves, and perhaps upregulation of this protein activated the antioxidative system in the leaves in response to LK stress, similar to that which occurred for OsSIK1. In plants, the phospholipase signaling pathway is also necessary under stress conditions. Phospholipase A (PLA) proteins constitute an important group of enzymes responsible for phospholipid hydrolysis in lipid signaling. PLA was up-regulated in the leaves; the high abundance of this protein was possibly a reason for the strong LK tolerance of alligator weed. Laohavisit *et al*. reported that annexin functions as a Ca^2+^-permeable channel in the plasma membrane to mediate radical-activated plasma membrane Ca^2+^- and K^+^-permeable conductance in root cells [35]. In our study, one annexin was up-regulated, and the other was down-regulated, suggesting that this family of proteins has various functions. Glycine-rich RNA-binding protein (GRP) functions in a manner similar to cold-shock proteins and has RNA chaperone activity in barley [36]. Further research suggested that this protein is a key molecular component of hormone-regulated development and regulates gene expression at multiple levels, such as by modifying mRNA alternative splicing as well as via export, translation, and degradation [37]. In our proteomic data, GRP2 was up-regulated, while GRP5 was down-regulated, and the expression levels of the two genes showed the same tendencies as they did according to the qRT-PCR results. GRP5 may be a negative regulator involved in the response to abiotic stress similar to ZjGRP [38], and determining the unique function of GRPs requires additional experiments. Teosinte branched 1-cycloidea-pcf (TCP) transcription factors constitute a group of proteins with a conserved DNA-binding and dimerization motif known as the TCP domain. TCP15 from *Arabidopsis* acts as a repressor of anthocyanin accumulation under high light intensity by modulating the expression of transcription factors involved in the induction of anthocyanin biosynthesis genes [39]. Subsequent research suggests that TCP15 modulates auxin and cytokinin levels, the immune response, and the cell cycle in *Arabidopsis* [40,41]. In our study, one TCP15 was down-regulated, which may affect the expression of many target genes involved in cell cycle progression and the hormone levels in alligator weed leaf; therefore, TCP15 and its target genes should be the research focus of LK stress in the future.

### 3.3. DAPs Related to the Transport Process

The ABC transporter protein superfamily has the ability to hydrolyze adenosine triphosphate (ATP), which plays a vital function in DNA repair, RNA translocation, and the active transport of a wide variety of substrates across various types of membranes in cells. Research has suggested that the ABC family in rice functions in the enhancement of crop yield and stress tolerance [42]. Downregulation of ABC transporter family member 6 may have affected the bidirectionality transport of a large range of substrates (e.g., hormones and primary and secondary metabolites) to adapt to LK stress in the leaves in our study. However, further research is needed to clarify the details of its function. Zhou et al. reported that Patellin1 (PATL1) negatively modulates PM Na^+^/H^+^ antiport activity and modulates cellular redox homeostasis during salt stress in *Arabidopsis* [43]. In addition, PATLs are involved in auxin signaling cascades by influencing PIN auxin transporter relocation [44]. In our study, one PATL was up-regulated, and this protein may play a vital function in these two physiological processes in leaves. Glycosylation is a fundamental cellular process that occurs in the lumen of both the Golgi apparatus and endoplasmic reticulum. Sugar transporters are an essential component of the glycosylation pathway [45], and overexpressing DsSWEET12 (sugar transporter) in *Arabidopsis* resulted in higher tolerance to osmotic and oxidative stresses [46]. Loss-of-function mutation of AtSUC2 and AtSUC4 led to higher sucrose contents in shoots and lower sucrose contents in roots [47]. In our study, the sugar transporter ERD6-like 6 (ERD6) was up-regulated, so this transporter may transport more sugar to the roots to induce lateral root growth, helping alligator weed improve LK tolerance. Plant nonspecific lipid transfer proteins (nsLTPs) are low-molecular-mass basic proteins belonging to the plant specific prolamin superfamily. SiLTP-overexpressing *Setaria italica* exhibited improved tolerance under salt and drought stresses, and its transcription is regulated by the transcription factor ABA-responsive DRE-binding protein [48]. In our study, one nsLTP was up-regulated, and it is vital to identify related transcription factors to elucidate the function of this protein in LK stress.

### 3.4. ApCPX1 Played Positive Roles in LK Stress

Peroxidases (PODs) are very important enzymes that are crucial for various biological processes in cells. According to their different isoelectric points, peroxidases can be divided into anion, neutral, and cation peroxidases. Many plant cation peroxidases have been shown to catalyze the oxidative decarboxylation of IAA to form indole-3-methanol or 3-methyleoxindole [49]. Three cationic peroxidases (AtPrx2, AtPrx25, and AtPrx71) catalyze lignin biosynthesis in *Arabidopsis* [50]. In our study, overexpression of *ApCPX1* in transgenic tobacco could increase LK stress tolerance. Our results suggested that the seed germination rate, POD activity, and K^+^ content increased and that the H_2_O_2_ content decreased in the transgenic tobacco lines compared with the WT under LK stress (Figure 7). ApCPX1 function was different from that of RCI3 (a peroxidase) in *Arabidopsis*. Overexpressing *RCI3* plants result in more ROS production and improved *AtHAK5* expression in response to LK stress [17]. Yang *et al*. reported overexpression of OsHAK5 increased the net K influx rate about 2.6-fold compared with that in the wild type under LK stress in rice root [51]; in our study, net K efflux rates were considerably lower in transgenic roots than in those of the WT under LK stress (Figure 8D). Similar results were also observed in a previous study; Li *et al.* reported in the oocytes expressing NRT1.5, the K^+^ efflux rates were completely depressed, suggesting that NRT1.5 can mediate K^+^ efflux out of cells [52]. Exposure of plants to LK stress leads to the oxidative damage initiated by reactive oxygen species (ROS) and ROS-induced potassium efflux in *Arabidopsis* roots. ROS can activate GORK channels to promote potassium outflow from cells [53]. These findings suggest that *ApCPX1* overexpression may clear excess ROS and depress GORK channel activity to decrease K efflux under LK stress. More experiments are needed to prove which potassium transporter or channel works, so the molecular mechanism of ApCPX1 involved in LK stress in leaves will need to be studied in the future; in this way, the reason for the high LK tolerance of alligator weed can be explained comprehensively.

## 4. Materials and Methods

### 4.1. Physiological Experiments on Alligator Weed Leaves

Shoots of naturally growing alligator weeds were collected from a test field of Sichuan Agricultural University (Chengdu, China) and cultured hydroponically in a growth chamber for 10 days to induce root growth. The greenhouse was maintained under a 16 h/8 h day/night light cycle and a 28 °C/25 °C day/night temperature cycle. The nutrient solution was replenished every two days and was prepared as described previously [13]. The alligator weed had developed strong roots after 10 days of culture. Half of the plants were then transferred to a low-potassium nutrient solution (lacking K_2_SO_4_); these plants were considered the LK samples. As the CK samples, the other half continued to grow in the solution with 1 mM K^+^. Leaf samples of the LK and CK were then collected after 15 days of LK treatment for physiological and molecular measurements. The total chlorophyll content, SOD activity, and soluble protein content were measured as described previously [54,55]. The net photosynthetic rate was analyzed on the 15th day of treatment using a portable photosynthesis meter (Li-6400, LI-COR, Lincoln, NE, USA) between 9:00 and 11:00 a.m. according to described previously methods [56].

### 4.2. Protein Extraction and TMT Labeling

Six leaf samples were ground to a powder in liquid nitrogen and then transferred to a 5-mL centrifuge tube. The protein extraction followed a previously described protocol [54], and the protein concentration was determined with a BCA kit according to the manufacturer’s instructions. For digestion, the protein solution was reduced with 5 mM dithiothreitol for 30 min at 56 °C and subsequently alkylated with 11 mM iodoacetamide for 15 min at room temperature in the dark. The protein sample was then diluted by adding 100 mM triethyl-ammonium bicarbonate buffer (TEAB) to obtain a urea concentration less than 2 M. Finally, trypsin was added at a 1:50 trypsin:protein mass ratio for the first digestion overnight and at a 1:100 trypsin:protein mass ratio for a second 4 h digestion. After trypsin digestion, the peptides were desalted using a Strata X C18 SPE column (Phenomenex, Torrance, CA, USA and vacuum dried. The peptides were reconstituted in 0.5 M TEAB and processed according to the manufacturer’s protocol for the TMT kit.

### 4.3. HPLC Fractionation and LC–MS/MS Analysis

The tryptic peptides were fractionated by high pH reverse-phase HPLC using an Agilent 300 Extend C18 column (5 μm particles, 4.6 mm ID, and 250 mm length). The tryptic peptides were dissolved in 0.1% formic acid (solvent A) and directly loaded onto a custom-made reversed-phase analytical column. The gradient involved an increase from 7% to 25% solvent B (0.1% formic acid in 98% acetonitrile) for 38 min and from 25% to 40% for 14 min, after which it increased to 80% for 4 min and then was maintained at 80% for the last 4 min, all at a constant flow rate of 700 nL/min on an EASY-nLC 1000 UPLC system. The peptides were subjected to an NSI source followed by tandem mass spectrometry (MS/MS) by using a Q Exactive^TM^ Plus system (Thermo, Waltham, MA, USA) coupled online to the UPLC. The electrospray voltage applied was 2.0 kV. The *m*/*z* scan range was 350 to 1000 for the full scan, and the intact peptides were detected by the orbitrap instrument at a resolution of 70,000. The peptides were then selected for MS/MS using the NCE setting at 27, and the fragments were detected by the orbitrap instrument at a resolution of 17,500. The maximum IT was set at 20 s for the full MS and to “auto” for MS/MS. The isolation window for MS/MS was set at 2.0 *m*/*z*.

### 4.4. Database Search and DAP Functional Analysis

The resulting MS/MS data were processed using the Maxquant search engine (v.1.5.2.8,Matrix Science Inc., Boston, CA, USA). The tandem mass spectra were searched against alligator weed transcription data. The mass tolerance for the precursor ions was set as 20 ppm in the first search and 5 ppm in the main search, and the mass tolerance for the fragmented ions was set as 0.02 Da. Carbamidomethyl of Cys was specified as the fixed modification, and oxidation of Met was specified as the variable modification. The FDR was adjusted to <1%, and the minimum score for the peptides was set to >40. The DAPs were assigned to the NCBI nonredundant (Nr) protein database using the Blast2GO program to obtain their functional annotation. The Gene On-tology (GO) annotation proteome was derived from the UniProt-GOA database (available online: http://www.ebi.ac.uk/GOA/), and the Kyoto Encyclopedia of Genes and Genomes (KEGG) database was used to annotate the protein metabolic pathways. DAP subcellular localization prediction was conducted followed by TargetP1.1 (available online: http://www.cbs.dtu.dk/services/TargetP/).

### 4.5. PRM–MS Analysis

The changes in protein abundance obtained using the proteomic analysis were confirmed by a PRM–MS analysis carried out at Jingjie PTM-Biolab Co., Ltd. (Hang Zhou, China). The proteins (60 μg) from the leaf samples were prepared, reduced, alkylated, and digested with trypsin following the protocol for the TMT analysis. The obtained peptide mixtures were introduced into a mass spectrometer via a C18 trap column (0.10 × 20 mm; 3 μm) and then via a C18 column (0.15 × 120 mm; 1.9 μm). The raw data obtained were then analyzed using Proteome Discoverer 1.4 (Thermo Fisher Scientific, Waltham, MA, USA ). The FDR was set to 0.01 for the proteins and peptides. Skyline 2.6 software (downloaded from the MacCoss Laboratory at the University of Washington) was used for quantitative data processing and proteomic analysis.

### 4.6. qRT-PCR Analysis of the Gene Expression Level

For the qRT-PCR analysis, total RNA was extracted from the leaves using TRIzol reagent (Invitrogen, Carlsbad, CA, USA). The isolated total RNA was used to generate cDNA with a reverse transcriptase kit (Thermo, Tokyo, Japan). The relative quantification of the candidate genes by qRT-PCR was carried out using a 7500 Real Time PCR System (Bio-Ras, State of California, USA Life Technologies, San Francisco., CA, USA) following the manufacturer’s protocols. The formula 2^−ΔΔ*C*t^ was used to calculate the relative gene expression levels. Actin2/8 expression was used as the internal control, and three replicates were included. All the data are shown as the means ± SDs (*n* = 3). The primer sequences for the eight genes are listed in Table S3.

### 4.7. Experiments Related to Transgenic Tobacco

The coding DNA sequence (CDS) of *ApCPX1* from leaves after LK treatment was amplified by RT-PCR. The sequence of *ApCPX1* and the qRT-PCR primer sequences were listed (Supplementary material 4), and this gene was fused to a pBI121 plant transformation plasmid, which was then transformed into *Agrobacterium* GV3101. For gene transformation, tobacco (*Nicotiana tabacum* cv. K326) seeds were surface sterilized with 75% ethanol and 10% bleach for 5 and 15 minutes, respectively. The seeds were then washed with sterilized water for more than 10 minutes and sown in Murashige and Skoog (MS) agar medium in a growth chamber at 26 °C and under a light intensity of 200 μmol·m^−2^·s^−1^ with a 16 h light/8 h dark cycle. After one month, transformation was performed using the leaf disc cocultivation method. The transgenic plants were selected for kanamycin resistance and were verified by qRT-PCR. For the LK treatment, the surface-sterilized tobacco seeds were grown on two filter papers soaked in 1 mM K^+^ or 10 µM K^+^ solution for 10 days in a greenhouse at 26 °C under a 16/8 h day/night light cycle with 200 μmol·m^−2^·s^−1^ irradiance, after which they were harvested and used for physiological index measurements. The POD activity and H_2_O_2_ content were measured as described previously [55]. A stereomicroscope of the Leica company was used to observe tobacco root hairs; root samples were obtained from tobacco seedings growing on MS and LK media for 10 days. Net K^+^ fluxes were measured under the experimental conditions for 5 min to decrease variability due to fluctuations. Roots from at least six individual plants were measured in an independent experiment. Each plant was measured once. The measuring solution for K^+^ contained (in mM) 88.95 NaCl, 0.05 KCl, 2.4 NaHCO_3_, 0.71 CaCl_2_, 0.82 MgSO_4_·7H_2_O, and 15 MES/HEPES, pH 5.5/7.4.The measurements were carried out using the SIET system BIO-003A (Younger USA Science and Technology) at Xu-Yue Science and Technology (http://www.xuyue.net).

### 4.8. Statistical Analysis

For all generated data, at least three biological replicates were included. The data were subjected to unpaired Student’s *t* -tests at levels of *p* ≤ 0.01 and *p* ≤ 0.05. The data are shown as the means ± SEs (*n* = 3). Excel and the SPSS 14.0 statistical software package were used for the statistical analyses of the data.

## 5. Conclusions

Alligator weed is reported to have a strong ability to adapt to LK stress. In this study, physiological and quantitative proteomic changes in alligator weed leaves in response to LK stress were investigated. Total chlorophyll content, SOD activity, and net photosynthetic rate increased after treatment, KEGG analysis suggested that most DAPs were associated with secondary metabolism, the stress response, photosynthesis, protein synthesis, and degradation pathway. Further research suggested that overexpression of *ApCPX1* increased the LK stress tolerance of transgenic tobacco, the seed germination rate, POD activity, and K^+^ content increased, while the H_2_O_2_ content and net K efflux rates decreased in the transgenic tobacco lines compare to the WT after treatment. These results provided valuable information for the adaptive mechanisms of alligator weed under LK stress and would help identify vital functional genes to apply to the molecular breeding in the future.

## Figures and Tables

**Table 1:** Confirmation of DAPs in proteomic analysis using PRM analysis.

Description	Change in TMT	*p*-Value of TMT	Change in PRM	*p*-Value of RPM
Malate synthase	1.31	0.00772	1.6	0.0004
Cysteine proteinase RD21A	1.4	0.00878	1.57	0.022
Betaine aldehyde dehydrogenase	0.82	0.0431	0.71	0.009
Antiviral protein MAP	0.83	0.00814	0.67	0.0011
Major latex protein 31	0.74	0.0315	0.63	0.005
Choline monooxygenase	0.69	0.0000391	0.61	0.002

## Supplementary Materials

Supplementary materials can be found at https://www.mdpi.com/1422-0067/21/7/2537/s1.
