# Supplementary figures and images for: Quantitative Proteomic Analysis of Alligator Weed Leaves Reveals That Cationic Peroxidase 1 Plays Vital Roles in the Potassium Deficiency Stress Response
Source: Int J Mol Sci. 2020 Apr 6;21(7):2537. doi: 10.3390/ijms21072537 (PMC7177825; doi:10.3390/ijms21072537)

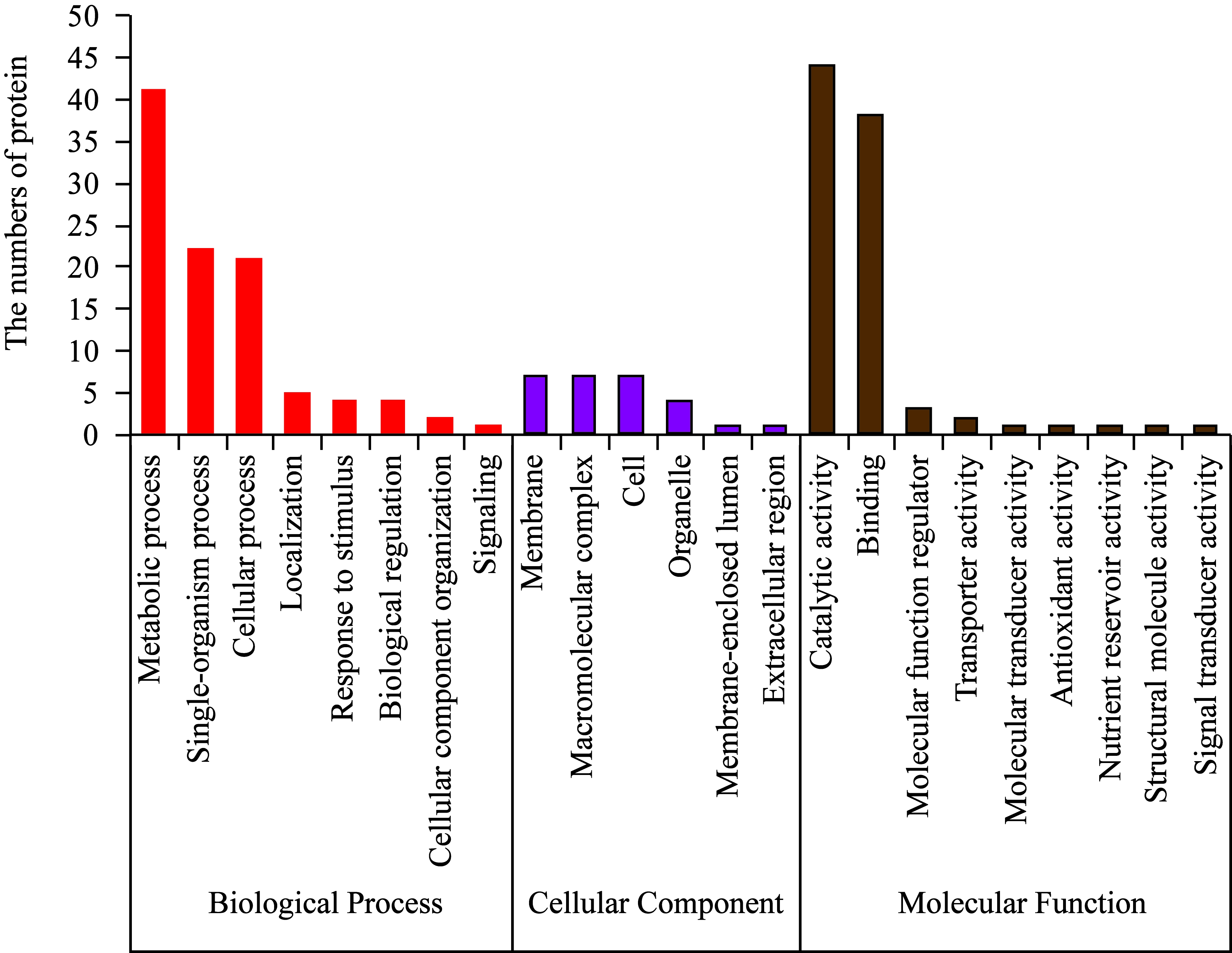

Supplement: Supplementary file 1 [file ijms-21-02537-s001.zip › Supplementary material/Figure S1.jpg]

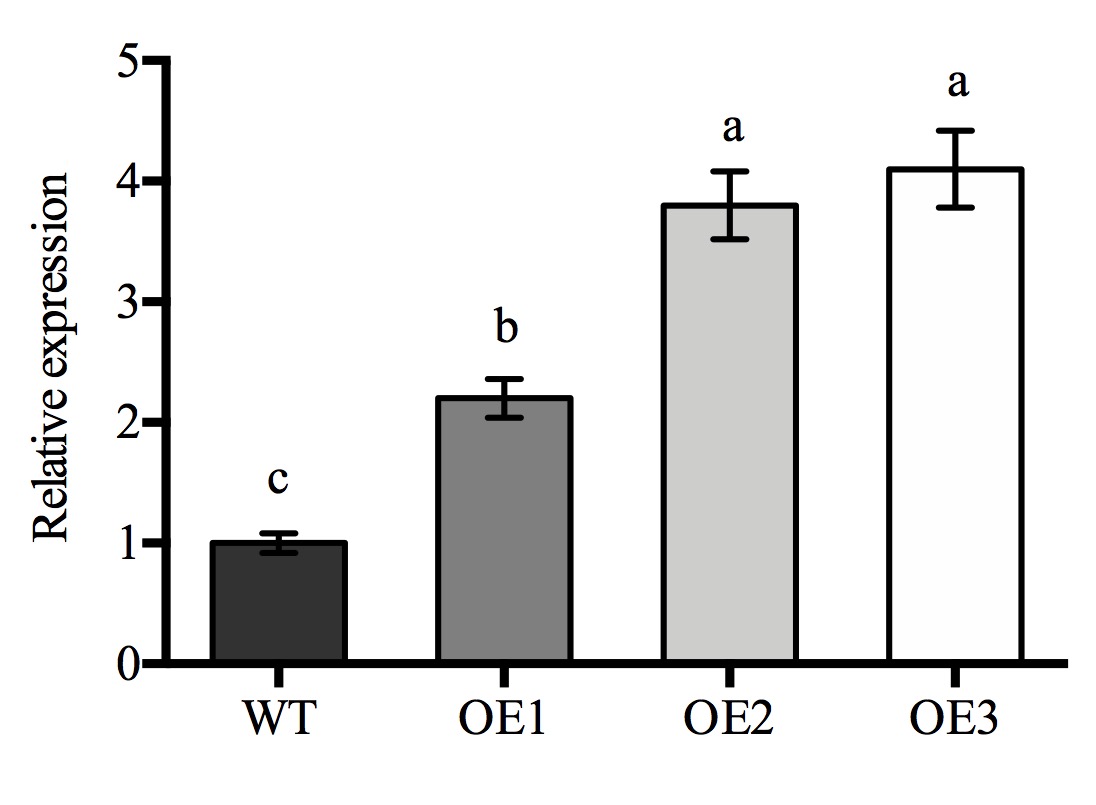

Supplement: Supplementary file 1 [file ijms-21-02537-s001.zip › Supplementary material/Figure S2.jpg]
